# Editorial: Vaginal dysbiosis and biofilms

**DOI:** 10.3389/fcimb.2022.976057

**Published:** 2022-08-09

**Authors:** António Machado, Claudio Foschi, Antonella Marangoni

**Affiliations:** ^1^ Universidad San Francisco de Quito USFQ, Colegio de Ciencias Biológicas y Ambientales COCIBA, Instituto de Microbiología, Laboratorio de Bacteriología, Quito, Ecuador; ^2^ Microbiology, Department of Experimental, Diagnostic and Specialty Medicine, University of Bologna, Bologna, Italy

**Keywords:** biofilms, vaginal dysbiosis, reproductive health, vaginal microbiota, accurate diagnostics, novel treatments

The vaginal microbiota is made up of a diversity of microorganisms (Pacha-Herrera et al.; Salinas et al., 2020), where commensal *Lactobacillus* species act as the first defense mechanism against the establishment of vaginal dysbiosis and vaginitis (Petrova et al., 2017) . When this balanced microbiota gets disrupted, the vaginal epithelium is less protected, and vaginal dysbiosis can succeed (**Figure 1**). It is characterized by a shift in microbial communities that include a progressive replacement of certain *Lactobacillus* species by pathogenic/opportunistic microorganisms (Machado and Cerca, 2015). This microbial shift can lead to bacterial vaginosis (BV), vulvovaginal candidiasis (VVC), aerobic vaginitis (AV), among others. These vaginal dysbioses are characterized by an overgrowth of multiple pathogens and promoting mixed infections. Another fact is the ability of certain pathogens to develop biofilms (Machado and Cerca, 2015; Castro et al.). Biofilms represent the predominant mode of microbial growth in nature, leading to important public health problems, as infections and negative interactions affecting the host immune system and the reproductive health outcomes in women (Machado et al., 2017). Vaginal dysbiosis is also associated with an increased risk of acquiring human immunodeficiency virus (HIV), Herpes simplex type 2, and other sexually transmitted infections, as Chlamydia (Parolin et al., 2018; Ceccarani et al., 2019). Understanding these vaginal microbiota dynamics is the key to developing accurate diagnostics and novel treatments. Due to the heterogeneity of species within biofilms, it has been difficult to assess the relevance of individual species to the pathogenesis of vaginal dysbiosis.

**Figure 1:**
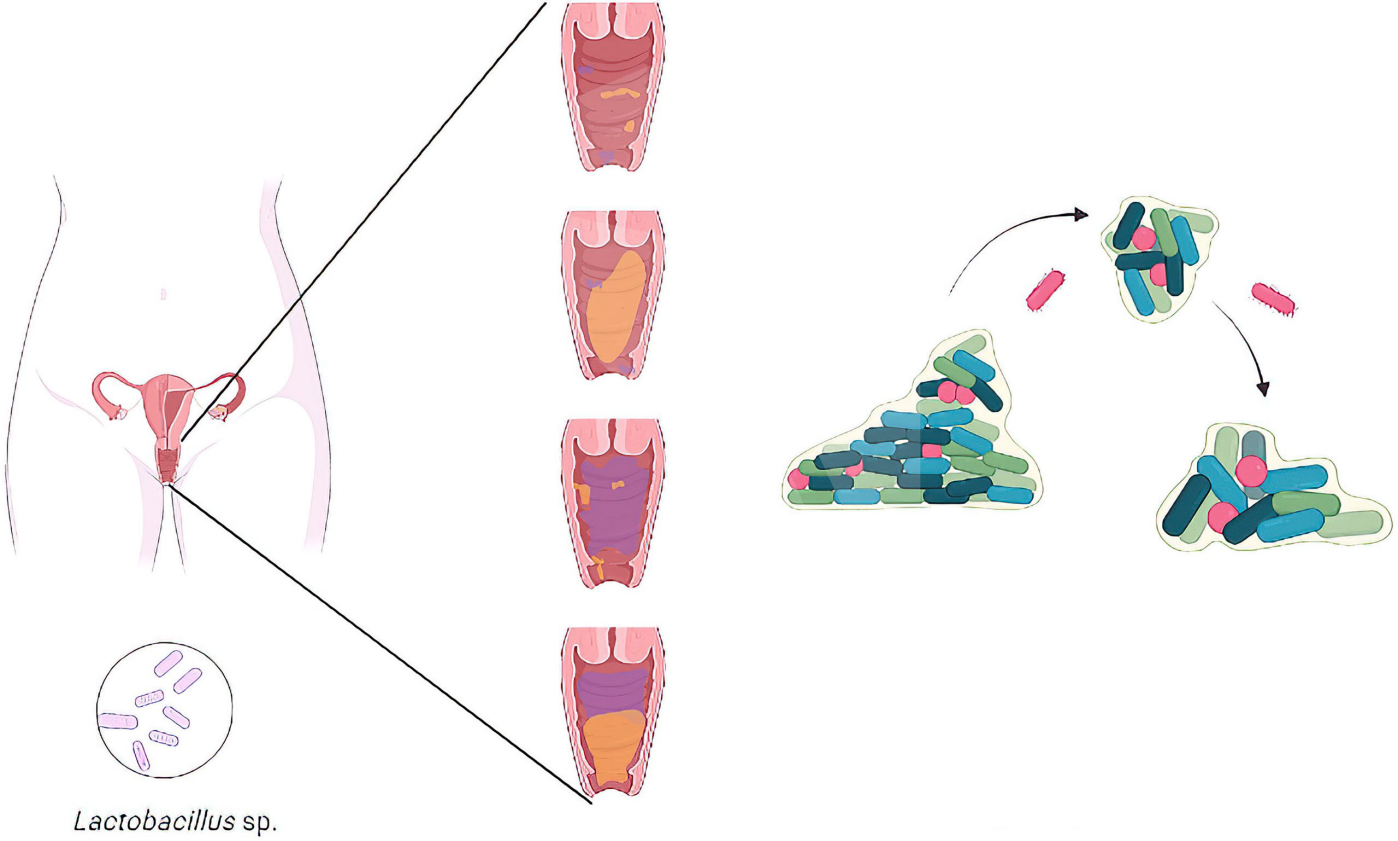
Representative illustration of the transition from a healthy vaginal microbiota with a high concentration of Lactobacillus species to the establishment of a vaginal dysbiosis in women.

This Research Topic focuses on several factors associated with vaginal dysbiosis and biofilm development. The first article reported the development of a novel peptide nucleic acid (PNA) probe targeting *Fannyhessea (Atopobium) vaginae* and validated with another *Gardnerella-*specific PNA probe. The authors showed a possible method for BV diagnosis evidencing excellent sensitivity and specificity for *F. vaginae*-*Gardnerella* biofilms (Sousa et al.). The second article by Zheng et al. reviewed the role of *Lactobacillus iners*, postulating its role as transitional species that colonizes after the vaginal microbiota is disturbed. *L. iners* offers an overall less protection against vaginal dysbiosis and leads to BV. Under certain conditions, *L. iners* is a genuine vaginal symbiont, but it can also act as an opportunistic pathogen.

Castro et al. evidenced that the crystal violet (CV) staining method, despite its widespread utilization, fails to properly quantify multiple species in BV-related biofilms, more exactly *Gardnerella vaginalis*, *F. vaginae*, and *Prevotella bivia*. Meanwhile, Ferreira et al. discussed sialidase activity in the cervicovaginal fluid (CVF) and its association with microscopic findings of BV. Through sequencing bacterial 16S rRNA gene in 140 vaginal samples, the authors demonstrated that 44 participants (31.4%) had molecular-BV, of which 30 (68.2%) had sialidase activity, suggesting that sialidase activity in molecular-BV is associated with changes in bacterial components of the microbiome. In the fifth article, Costantini et al. showed that vaginal microbiota dominated by lactobacilli protects women from sexually transmitted infections, in particular HIV type 1 (HIV-1), and partially this protection is mediated by *Lactobacillus*-released extracellular vesicles (EVs). These authors found that EVs released by lactobacilli protect human cervico-vaginal tissues *ex vivo* and isolated cells from HIV-1 infection by inhibiting HIV-1-cell receptor interactions. Also, they identified numerous EV-associated proteins involved in this protection.

Qin and Xiao reviewed the new genotyping of *Gardnerella* species (*G. leopoldii*, *G. piotii*, and *G. swidsinskii*) describing the genetic diversity when compared with *G. vaginalis* and reporting new findings on the correlation with BV. Furthermore, Xiao et al. reported 48 symptomatic patients with clinical diagnoses of VVC complicated with BV and the results obtained on their treatments with oral metronidazole combined with local clotrimazole, assessing the drug efficacy and vaginal microbiome alterations. Their results evidenced significant alterations on vaginal microbiome in BV+VVC mixed vaginitis patients and an enhanced treatment for BV. It is worth to be underlined that this is the first study to investigate multiple characteristics of the vaginal microbiome in patients with BV+VVC before and after drug treatment.

On the other hand, Pacha-Herrera et al. evaluated the probiotic activity promoted by individual and multi-microbial consortia of five vaginal lactobacilli (*Lactobacillus iners*, *Lactobacillus crispatus*, *Lactobacillus gasseri*, *Lactobacillus jensenii*, and *Lactobacillus acidophilus*) among healthy women and women with BV or AV. The qualitative analysis through PCR assays was realized on 436 vaginal samples from a previous study (Salinas et al., 2020) and statistical analysis evaluated associations between lactobacilli and vaginal microbiota. Multi-microbial clustering model was also realized to determine the probiotic relationship between lactobacilli and vaginal dysbiosis. Concerning the individual effect, *L. acidophilus*, *L. jensenii*, and *L. crispatus* showed the highest normalized importance values against vaginal dysbiosis. *L. acidophilus* showed a significant prevalence on healthy microbiota against both dysbioses (BV, *p* = 0.041; and AV, *p* = 0.045). *L. jensenii* only demonstrated significant protection against AV (*p* = 0.012). Finally, the study evidenced a strong multi-microbial consortium by *L. iners*, *L. jensenii*, *L. gasseri*, and *L. acidophilus* against AV (*p* = 0.020) and BV (*p* = 0.009), lacking protection in the absence of *L. gasseri* and *L. acidophilus* (Pacha-Herrera et al.).

Last, but not least, in the ninth article, Swidsinski et al. analyzed different types of clue cells (epithelial cells heavily covered with adherent bacteria), which are accepted as a key clue to BV diagnosis. The authors investigated adhesive and cohesive patterns of main microbiota groups in vaginal discharge using fluorescence *in situ* hybridization (FISH) on BV samples of 500 women. FISH analysis evaluated the spatial distribution of BV-related bacterial groups. The authors evidenced four patterns, such as dispersed (non-adherent bacteria), dispersed adherent bacteria, cohesive (non-adherent) bacteria, and cohesive adherent bacteria. Direct cohesive adherence to the epithelial cells representing true clue cells was unique for *Gardnerella* species. The study illustrated that taxon indifferent imaging is inadequate for the correct BV diagnosis, being BV constituted by a mix of at least two different conditions, more exactly biofilm vaginosis and bacterial excess vaginosis.

## Author contributions

AMac wrote the first draft. CF, and AMar provided critical comments and editorial suggestions for revisions. All the authors agreed on the submitted version.

## Acknowledgments

AMac would like to thank all the staff of the Microbiology Institute of USFQ and COCIBA, as well as the Research Office of Universidad San Francisco de Quito for their continuous research support. Likewise, AMac wishes to thank CF and AMar for accepting to collaborate on this Research Topic in Frontiers and for their expertise acknowledge during the editorial process. Finally, AMac also thanks Robert Josue Rodríguez-Arias for creating the figure for this editorial article.

## Conflict of interest

The authors declare that the research was conducted in the absence of any commercial or financial relationships that could be construed as a potential conflict of interest.

## Publisher’s note

All claims expressed in this article are solely those of the authors and do not necessarily represent those of their affiliated organizations, or those of the publisher, the editors and the reviewers. Any product that may be evaluated in this article, or claim that may be made by its manufacturer, is not guaranteed or endorsed by the publisher.
